# Using comparative genomics to reorder the human genome sequence into a virtual sheep genome

**DOI:** 10.1186/gb-2007-8-7-r152

**Published:** 2007-07-30

**Authors:** Brian P Dalrymple, Ewen F Kirkness, Mikhail Nefedov, Sean McWilliam, Abhirami Ratnakumar, Wes Barris, Shaying Zhao, Jyoti Shetty, Jillian F Maddox, Margaret O'Grady, Frank Nicholas, Allan M Crawford, Tim Smith, Pieter J de Jong, John McEwan, V Hutton Oddy, Noelle E Cockett, the International Sheep Genomics Consortium

**Affiliations:** 1 CSIRO Livestock Industries, Carmody Road, St Lucia, Queensland 4067, Australia; 2 SheepGenomics, L1, Walker Street, North Sydney, New South Wales 2060, Australia; 3 The Institute for Genomic Research, Rockville, Maryland 20850, USA; 4 BACPAC Resources, Children's Hospital Oakland Research Institute (CHORI), Oakland, California 94609, USA; 5 Department of Veterinary Science, The University of Melbourne, Parkville, Victoria 3010, Australia; 6 Centre for Advanced Technologies in Animal Genetics and Reproduction (ReproGen), University of Sydney, Werombi Road, Camden, New South Wales 2570, Australia; 7 AgResearch, Invermay Agricultural Centre, Puddle Alley, Private Bag 50034, Mosgiel 9053, New Zealand; 8 US Department of Agriculture, Agricultural Research Service, Northern Plains Area, Roman L Hruska US Meat Animal Research, P.O. Box 166, Clay Center, Nebraska 68933, USA; 9 Meat and Livestock Australia, 165 Walker Street, North Sydney, New South Wales 2059, Australia; 10 University of New England, Armidale, New South Wales 2351, Australia; 11 Utah State University, Logan, Utah 84322-4800, USA

## Abstract

**Background:**

Is it possible to construct an accurate and detailed subgene-level map of a genome using bacterial artificial chromosome (BAC) end sequences, a sparse marker map, and the sequences of other genomes?

**Results:**

A sheep BAC library, CHORI-243, was constructed and the BAC end sequences were determined and mapped with high sensitivity and low specificity onto the frameworks of the human, dog, and cow genomes. To maximize genome coverage, the coordinates of all BAC end sequence hits to the cow and dog genomes were also converted to the equivalent human genome coordinates. The 84,624 sheep BACs (about 5.4-fold genome coverage) with paired ends in the correct orientation (tail-to-tail) and spacing, combined with information from sheep BAC comparative genome contigs (CGCs) built separately on the dog and cow genomes, were used to construct 1,172 sheep BAC-CGCs, covering 91.2% of the human genome. Clustered non-tail-to-tail and outsize BACs located close to the ends of many BAC-CGCs linked BAC-CGCs covering about 70% of the genome to at least one other BAC-CGC on the same chromosome. Using the BAC-CGCs, the intrachromosomal and interchromosomal BAC-CGC linkage information, human/cow and vertebrate synteny, and the sheep marker map, a virtual sheep genome was constructed. To identify BACs potentially located in gaps between BAC-CGCs, an additional set of 55,668 sheep BACs were positioned on the sheep genome with lower confidence. A coordinate conversion process allowed us to transfer human genes and other genome features to the virtual sheep genome to display on a sheep genome browser.

**Conclusion:**

We demonstrate that limited sequencing of BACs combined with positioning on a well assembled genome and integrating locations from other less well assembled genomes can yield extensive, detailed subgene-level maps of mammalian genomes, for which genomic resources are currently limited.

## Abstract 2

Using BAC-end sequences, a sparse marker map and the sequences of the human, dog and cow genomes, an accurate and detailed sub-gene level map of the sheep genome has been constructed.

## Background

Sheep are a major farmed species, producing meat, pelts, and wool. They are closely related to cattle, which is both an advantage and a disadvantage for researchers. Internationally, substantial genomics research is being conducted in cattle, with a large number of cow expressed sequence tag sequences deposited in public databases, and a draft assembly of the cow genome sequence is available [1,2]. However, the downside is that although the sheep genome sequence would be of great benefit to the sheep research community, the sheep has not yet been prioritized by funding agencies for whole genome sequencing. In the meantime, the sheep genomics research community must identify the most efficient way to utilize the small amount of sheep sequence and to exploit investment in the other mammalian genomes, while laying the ground work for the eventual sequencing of the sheep genome itself.

The first version of the sheep linkage map, released in 1995, contained 246 markers and covered 2,070 cM [3]. The map was updated in 1998 [4] and in 2001 [5]. It has continued to be refined, and the current version (4.6) contains 1,374 markers from 1,333 loci covering 3,630 cM (Maddox JF and coworkers, unpublished data). The addition of markers to the map has been slow, and the focus has changed from microsatellites to single nucleotide polymorphisms (SNPs) and expressed sequence tags in order to benefit from new genotyping technology, as well as to allow easier cross-species genomic comparisons. In addition, a few hundred markers have been positioned using cytogenetic approaches, and there are a number of whole genome chromosome painting datasets for sheep [6,7]. In the main, each of these approaches provides support for the same high-level comparative map between sheep and human chromosomes. However, the current sheep maps lack the resolution required for effective use of modern genomics tools, such as SNP-based whole genome scans.

With the availability of the cow genome sequence, low coverage survey sequencing of the sheep genome combined with radiation-hybrid mapping is an attractive option [8,9]. This can be undertaken via whole genome shotgun sequencing of small or large insert libraries. The use of large insert libraries, such as bacterial artificial chromosomes (BACs), has the advantage of producing a physical resource that can be used for other experimental procedures, such as BAC-based genome sequencing. Fingerprinting of BAC libraries by restriction enzyme cleavage and the generation of BAC contigs based on analysis of overlapping fingerprints generates a series of contiguous segments of the genome being analyzed [10]. This is also frequently the next step in a large genome sequencing project. By assigning markers to BACs, and therefore to BAC contigs, the segments can be positioned on a genetic map [11]. The addition of end sequence data from the BACs can aid substantially in extending the BAC contigs [12]. This approach was used to construct a BAC based map of the cow genome [11]. Pooled genomic indexing of BACs combined with comparative genomics has been demonstrated [13], using part of a rhesus macaque BAC library with the human genome as the scaffold. Overgo probes designed against regularly spaced conserved regions of genomes have also been used to build BAC contigs [14,15]. Genome wide sets of universal probes have been designed [16], and in theory they could be used to construct whole genome contigs from large BAC libraries, with limited use of comparative genomics. However, BAC paired end mapping to the human, mouse, and rat genome sequences has been used to identify large scale rearrangements in the respective genomes [17]. This suggests that a similar approach could be applied to identify rearrangements in an organism with an unsequenced genome, if genome sequences from closely related species were available. The increasing number of complete and near complete genome sequences of other mammals suggests that a BAC end sequence and comparative genomics 'scaffolding' strategy might approach the resolution and accuracy of fingerprinting large BAC libraries.

For sheep the steps in such a comprehensive strategy would be to construct and end sequence a BAC library, and then to map the BAC ends to the cow, dog, and human genomes, and construct BAC-comparative genomic contigs (CGCs), which are then anchored and oriented using the current sheep maps. The construction of two small sheep BAC libraries has been described. However, coverage was only two [18] and three genome equivalents [19]. Although the optimal coverage for the scaffolding approach is unknown, the coverage provided by the smaller libraries is far too low. In addition, to overcome the limitations imposed by the potentially large number of BAC-CGCs, and the relatively sparse maps, other information will be required. End sequence profiling approaches developed for analysis of the rearrangements in human tumour genomes [20,21] may be applicable to the identification of rearrangements between the sheep and human genomes. In addition, the availability of complete genome sequences for a number of mammals has demonstrated that the mammalian genome consists of a large number of regions with no major rearrangements detected and regions with extensive rearrangements and broken synteny [17,22-25]. This information can be used to overcome the deficiencies, by enabling the linking of otherwise unconnected BAC-CGCs together. The resulting virtual sheep genome would enable the capture of the annotation of the human, dog, and cow genomes ordered appropriately for the sheep research community. A map of ordered sheep BAC end sequences would also be a useful tool for the development of further sheep genomics resources. Such resources could include an SNP chip covering the sheep genome derived from re-sequencing amplicons of the ordered BAC end sequences and a tiling path for sequencing the genome.

Here we describe the implementation of the BAC-CGC based approach using as much available information from the human, dog, and cow genome sequencing projects as possible, combined with all available sheep information and information on comparative vertebrate conserved synteny.

## Results

### Construction, characterization, and end sequencing of the sheep BAC library

**Figure 1:**
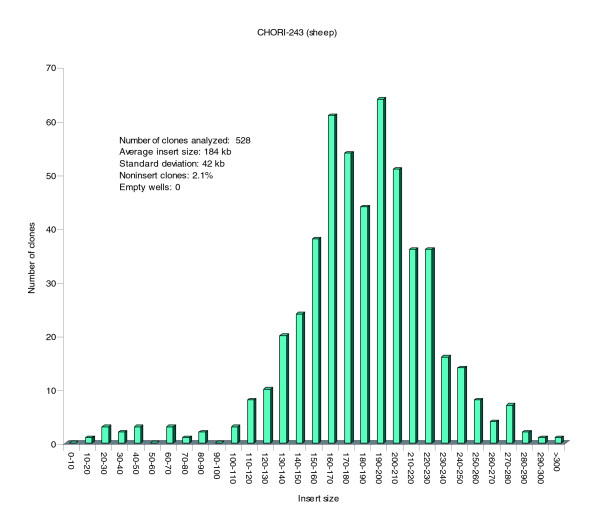
Distribution of insert size in the sheep BAC library CHORI-243. BAC, bacterial artificial chromosome; kb, kilobase.

A Texel ram was chosen as the source of the DNA for the construction of the BAC library, because the Texel is a popular terminal sire breed for meat production in several countries, and a Texel was the paternal grandsire breed of the sheep international mapping flock [3]. The particular animal used had accumulated about 8% inbreeding over the preceding five generations of matings (Smith T, unpublished data). The details of the library and construction are described under Materials and methods (see below), and the distribution of insert sizes of the first 528 clones is shown in Figure 1. Assuming a sheep genome size of 2.76 gigabases (the golden path length for the Btau2.0 assembly of the cow genome plus 5%), the 202,752 clones with an average size of 184 kilobases (kb) indicates that the library coverage is about 13.5-fold. End sequencing of the complete set of BAC clones yielded paired end reads for 179,047 (88%) clones (about 12.6-fold coverage of the sheep genome), single end reads for 13,951 (7%) clones, and no reads for 9,754 (4%) clones, giving a total of 372,045 BAC end sequences with a mean edited length of 687 bases. Including some multiple reads from the same end of a number of BACs, a total of 376,493 BAC end sequence reads were deposited in GenBank.

### Mapping sheep BACs to the cow, dog, and human genomes

Among the mammalian genomes with high sequence coverage, the cow is the most closely related species to the sheep. However, at the time of these analyses, the cow genome assembly was an early draft release (Btau2.0), comprising a large number of scaffolds, many of which were not assigned to chromosomes and hence were not ordered or oriented. The dog genome [26] is the next closest available and has a much higher level of assembly, but it is not as well annotated as the human genome [23]. On the basis of the integrity of assembly and extent of annotation [27], the human was chosen as the third comparison species.

**Figure 2:**
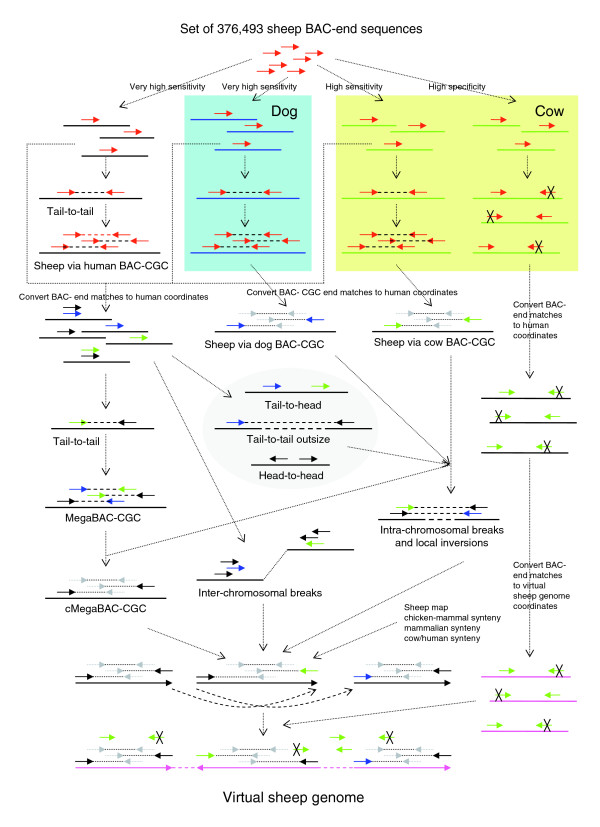
Data flow for the construction of the sheep virtual genome. Except where indicated in the colored boxes, all analyses were on the framework of the human genome. Sheep BAC end sequences are represented by short arrows, with the arrowheads located at the 3' end of the BAC end sequence. Paired ends are linked by dotted lines. Red arrows indicate where the sheep sequence was used in the analysis; the black (human), blue (dog), and green (cow) arrows indicate where the coordinates based on the respective genomes, or their conversion to the equivalent human or virtual sheep genome coordinates, were used in the analysis. The Xs represent BAC end sequences from the unpaired-in-cow group that have not been positioned anywhere, but are predicted to lie in the indicated position and orientation on the relevant genome. Gray arrows indicate the location of BAC end sequences within a BAC-CGC and are linked in pairs by dotted lines. The segments of framework genome, and the BAC-CGCs are colored as above and in addition pink for the virtual sheep genome. The arrowheads on the BAC-CGCs represent the orientation of the BAC-CGC relative to the human genome. Dashed lines with double headed arrows between segments of the human genome indicate path to create the virtual sheep genome. BAC, bacterial artificial chromosome; CGC, comparative genome contig.

The complete set of sheep BAC end sequences was aligned against each of the three sequenced genomes at high sensitivity. For each genome assembly, all aligned BACs with the two end sequences reading in opposing directions with internal 3' ends (tail-to-tail paired end BACs) between 10 and 500 kb apart on the same chromosome were identified and positioned on the relevant genome (Figure 2). As expected, because of the fragmented nature of the early draft assembly of the cow genome, the number of tail-to-tail paired end BACs was the lowest for this species (Table 1). Randomization of the mappings of the complete set of BAC end sequences to the human genome allowed us to estimate the false position rate to be 20 (0.024%) incorrectly assigned BACs. Given this very low error rate, BAC contigs were constructed with all overlapping aligned BACs, including regions with 1× coverage. BAC-CGCs were independently constructed on the genomes of all three species, and again the cow genome gave the largest number and the smallest average sized BAC-CGCs (Table 1).

**Table 1:** BAC-CGC construction results

Comparison genome	feature	Ovine BACs mapped tail-to-tail	Number of contigs	Mean span of contigs (Mb)	Total span of contigs (Gb)	Percentage of comparison genome^a^
Cow	BAC-CGCs	32,602	4,026	0.40	1.59	60.8%
Dog	BAC-CGCs	58,757	2,104	0.98	2.05	86.8%
Human	BAC-CGCs	52,338	2,447	0.96	2.35	82.7%
Human	MegaBAC-CGCs	84,624	1,257	2.04	2.57	90.5%
Human	Consolidated MegaBAC-CGCs	84,624	1,172	2.21	2.59	91.2%

^a^Golden path length of genome assemblies cow 2.62 Gb, dog 2.36 Gb (no Y chromosome in the golden path), and human 2.84 Gb (excludes the sequenced region of the Y chromosome). BAC, bacterial artificial chromosome; CGC, comparative genome contig; Gb, gigabases; Mb, megabases;

### Construction of MegaBAC-CGCs

The fragmented assembly of the early draft of the cow genome limits the benefits of the availability of a genome assembly from a closely related species. To maximize the utilization of the cow genome sequence, the coordinates of the BAC end mappings to the cow genome were transferred to the coordinate framework of the human genome (Figure 2). The coordinates of the BAC end mappings to the dog genome were also transferred to the framework of the human genome. Tail-to-tail paired end BACs between 10 and 500 kb apart were then identified, with the minimum requirement that one of the three possible positions on the human genome for each of the two BAC end sequences from each BAC contribute to the BAC location, irrespective of species. For example, the position of one end of the BAC could be derived from the original mapping of a BAC end to the cow genome, and the other end of the BAC could be derived from the original mapping to the dog genome (Figure 2).

**Figure 3:**
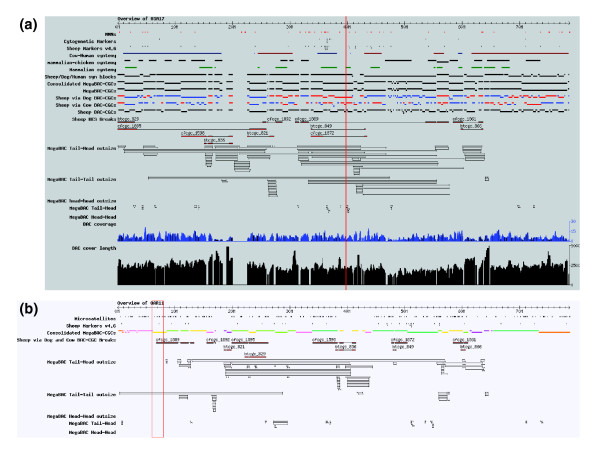
Representative chromosomes from the human and virtual sheep genome browsers. **(a) **Chromosome overview of HSA17 showing some of the datasets generated during the construction of the virtual sheep genome. The human genome browser overview tracks shown are as follows and are labelled as referred to in the text: unsequenced regions; gaps in the human genome assembly; cytogenetic markers; sheep markers version 4.6; cow-human conserved synteny; chicken-mammal conserved synteny; mammalian conserved synteny; sheep-dog-human conserved syntenic blocks calculated from the mapping of the sheep BACs to the dog and human genomes; consolidated MegaBAC-CGCs (the final set of 1,172 BAC-CGCs generated from the MegaBAC-CGCs) and the sheep-via-dog and sheep-via-cow BAC-CGCs; MegaBAC-CGCs (calculated from the MegaBAC analysis and before the final consolidation); sheep-via-dog BAC-CGCs (built on the dog genome and mapped onto the human genome); sheep-via-cow BAC-CGCs (built on the cow genome and mapped onto the human genome). **(b) **Chromosome overview of OAR11 showing cMegaBAC-CGC sections color coded to indicate the method and likely robustness of assignment (Table 3). A selection of virtual sheep genome browser overview tracks is shown. Labelling is as above; in addition, the microsatellite tracks are shown. BACs are shown in the tail-head outsize track, tail-tail outsize track, and so on, on the basis of their group in the MegaBAC analysis, not their actual size and BAC-end sequence orientations in the virtual sheep genome. All images are from genome databases displayed using Gbrowse [45]. BAC, bacterial artificial chromosome; CGC, comparative genome contig; HSA, human chromosome; OAR, sheep chromosome.

Using the complete set of BAC mappings, a new set of sheep BAC-CGCs mapped to the human genome was calculated: the MegaBAC-CGCs (Table 1). Randomization of the BAC end mapping data used for the construction of the MegaBAC-CGCs predicted a false position rate to be 47 (0.056%) incorrectly assigned BACs. This approach increased the size of the BAC-CGCs and almost halved the number obtained just from the direct mapping of sheep BAC end sequences to the human genome, but with only a small increase in genome coverage (Table 1). This set of MegaBAC-CGCs contained about 47.3% of the paired BAC end sequence reads as members of paired tail-to-tail BACs mapped to the human genome (about 5.4-fold average coverage), which represents a substantial increase over the direct human mapping alone (about 29.2%; Table 1). The BAC coverage was plotted along each chromosome (Figure 3a) and ranged up to 23-fold in some regions, with the average of coverage of human chromosomes (HSA) varying from 1.86-fold on the X chromosome and 2.5-fold on HSA22 (large regions of HSA22 are unsequenced) to 6.6-fold on HSA18. BAC average length was also calculated and overall was close to the library average length of inserts of 184 kb.

### Identification of intra-chromosomal breakpoints and local rearrangements

Since the current version of the sheep linkage map contains only 1,333 loci and there are 1,257 MegaBAC-CGCs, most of the BAC-CGCs could not be unequivocally positioned and oriented on the sheep map. In order to further reduce their number, and to identify breakpoints in each of the genomes relative to the sheep genome, the sheep BAC-CGCs constructed on the dog and cow genome frameworks were mapped across to the human genome (Figure 2). For each BAC-CGC from each species, the block(s) of BACs mapped to the relevant genome, and present within a single MegaBAC-CGC or across adjacent ones built on the human genome, were identified, and sets of sheep-via-dog and sheep-via-cow BAC-CGCs were constructed on the human genome (Figures 2 and 3a). The MegaBAC-CGCs on the human genome were then consolidated on the basis of the overlaps between the MegaBAC-CGCs and the sheep-via-dog and sheep-via-cow BAC-CGCs (Figure 2) to create 1,172 consolidated MegaBAC-CGCs (cMegaBAC-CGCs; Figure 3a).

To identify intra-chromosomal breakpoints present in the human genome and not in the sheep genome, but in one or both of the dog and cow genomes, we identified BAC-CGCs from the dog and cow genomes that contained blocks of BACs that mapped to two or more nonadjacent cMegaBAC-CGCs on the human genome (Figures 2 and 3a). Twenty such BAC-CGCs linking cMegaBAC-CGCs on the same chromosome were identified from the cow genome and 39 from the dog genome. The human cMegaBAC-CGCs were not consolidated further based on these data, but the linkages between the cMegaBAC-CGCS and their relative orientations in the human genome were recorded for use in the construction of the virtual sheep genome.

During the analysis it became clear that the BACs with tail-to-head paired ends (the reads from both ends aligned to the genome with the same orientation and in the range 10 to 500 kb) were not randomly positioned on the genome (Figure 3a). The predicted frequency of clustered tail-to-head BACs from the randomization analysis was zero, but 73% were in clusters of two or more BACs (Table 2). With an expected false position rate of about 2%, most of these BACs are likely to be correctly positioned. To further utilize the BAC end sequence mapping information, we undertook an end sequencing profile-type analysis [20,21] by extending our datasets to include all BACs with both ends mapped to the same chromosome, including the outsize BACs (the reads from both ends aligned to the genome closer together than 10 kb or further apart than 500 kb). Although a much smaller group than the tail-to-head BACs, the ends of the majority of head-to-head BACs were also clustered in the genome. With an estimated false position rate of about 10%, the majority of these BACs are also likely to be correctly positioned (Table 2). Unlike the ratio of tail-to-tail:tail-to-head:head-to-head BACs (345:10:1), the ratio of the equivalent sets of outsize BACs was close to the ratio expected from a random set of positions (1:2:1), with a slight over-representation of tail-to-head outsize BACs. In addition, a smaller proportion of the tail-to-tail outsize and head-to-tail groups, and even fewer head-to-head outsize BACs were located in clusters. This suggests that the actual false position rates in the groups of outsize BACs are likely to be quite high. The linkages between the cMegaBAC-CGCs predicted by these BACs and their relative orientations in the human genome were recorded for use in the assembly of the virtual sheep genome. To reduce the background of random mappings, only clustered outsize BACs were considered (Table 2).

**Table 2:** Size and composition of BAC groups in the MegaBAC analysis on the human genome and contribution to these groups by BACs present in the tail-to-tail groups from mapping onto the cow and/or dog genomes.

MegaBAC group	MegaBAC analysis			BACs in tail-to-tail group in cow and/or dog				
	
	Number of BACs	% False position^a^	Clustered (*n *[%])	% of MegaBAC group	Fold enrichment clustered	Cow or dog (*n*)	Dog (*n*)	Cow (*n*)
Tail-to-tail	84,624	0.056%	NA (NA)	78%	na	66,111	56,598	29,424
Tail-to-tail outsize	1,629	48%	451 (28%)	21%	2.4	350	195	202
Tail-to-head	2,477	2%	1,811 (73%)	36%	1	903	460	578
Tail-to-head outsize	4,134	38%	1,295 (31%)	18%	2	762	380	498
Head-to-head	245	10%	183 (75%)	7.8%	0.6	19	12	9
Head-to-head outsize	1,356	58%	47 (3.5%)	14%	0.7	197	63	159
Break	27,829	~100%	NA (NA)	9.4%	na	2,623	944	1,834
Unpaired	52,663^b^	na	NA (NA)	0.1%^c^	na	38	12	28
No hits	18,113	na	NA (NA)	na	na	0	0	0

^a^Calculated from randomisation of the MegaBAC analysis dataset. ^b^Includes 13,857 BACs with only one sequenced end. ^c^Percentage of BACs with both ends sequenced, but only one end positioned in MegaBAC analysis. BAC, bacterial artificial chromosome; NA, not applicable.

**Figure 4:**
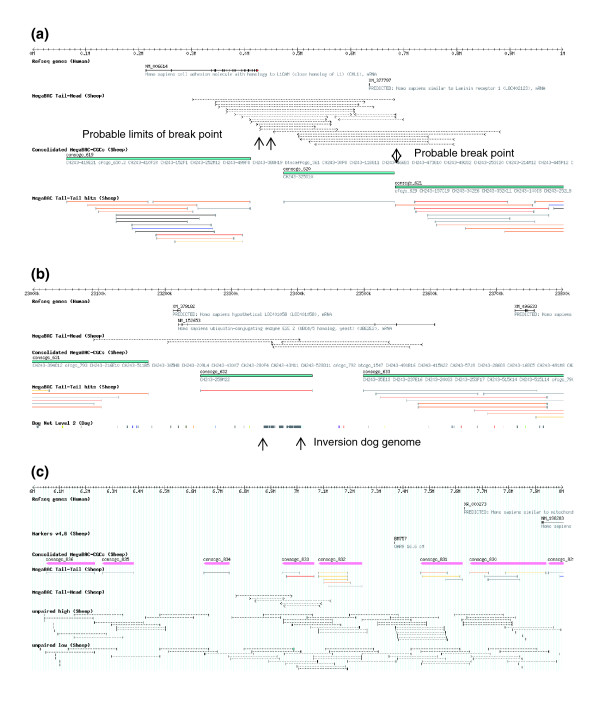
Examples of detailed views of the sheep BAC mapping information on the human and virtual sheep genomes. **(a) **A short section of human chromosome HSA3 from 0 to 1 Mb, showing human RefSeq genes, tail-to-head and tail-to-tail MegaBAC analysis BACs, and cMegaBAC-CGCs. **(b) **A short section of human chromosome HSA3 from 23 to 23.8 Mb, showing tracks as above and the Dog net level 2 track. **(c) **A region of low confidence in the virtual sheep genome. The region is from sheep chromosome OAR9. Tracks shown are the human RefSeq genes from NCBI, sheep markers from the Sheep Map version 4.6, the cMegaBACs, the tail-to-tail BACs from the MegaBAC analysis, and unpaired-in-cow, shown as high (single hits to the cow genome with the parameters used) and low (more than one hit to the cow genome with the parameters used) confidence sets. The dotted lines in the unpaired tracks indicate the predicted extent of the BACs in the genome assuming all BACs are 184 kb long (the average length of the BACs in the library). All images are from genome databases displayed using Gbrowse [45]. BAC, bacterial artificial chromosome; CGC, comparative genome contig; HSA, human chromosome; kb, kilobase; Mb, megabase; NCBI, National Center for Biotechnology Information; OAR, sheep chromosome.

Many of the clusters of tail-to-head and head-to-head BACs appear to identify the sites of local rearrangements in the sheep genome relative to the human genome. Indeed, the genome coverage of the BAC library is sufficiently high that the sites of some rearrangements can be mapped to very small regions of the genome. In an example, close to the beginning of HSA3, one end of an approximately 210 kb inversion appears to lie within a region of less than 30 kb and the other end in a region of less than 1.5 kb (Figure 4a). Both predicted breakpoints lie outside of known or predicted genes. Because the breakpoints lie outside of cMegaBAC-CGC 620, it was flagged for inversion during the construction of the virtual sheep genome. In a second example, further along HSA3, the smaller number of BACs involved appears at first sight to be less informative about the likely locations of the breakpoints (Figure 4b). However, inspection of the dog-to-human net tracks identified a small inversion in the dog genome relative to the human genome, which lies within cMegaBAC-CGC 632. The sheep tail-to-head BACs are consistent with the sheep genome containing the equivalent inversion. This rearrangement lies entirely within a large intron of the *UBE2E2 *gene and therefore it is predicted not to alter the structure of the final mRNA product. Because the rearrangement probably does not span cMegaBAC-CGC 632, it was not flagged for inversion during the construction of the virtual sheep genome.

We have not attempted to resolve all of the small rearrangements predicted by these groups of BACs. Rather, the display of the information on the genome browsers in the relevant tracks allows users to evaluate for themselves the possible nature and consequences of the rearrangements.

### Tail-to-tail BACs on cow and/or dog, but not in the MegaBAC analysis

Approximately 7% of the paired end tail-to-tail BACs mapped to the cow and/or dog genomes were not positioned on the human genome as paired end tail-to-tail BACs in the MegaBAC analysis (Table 2). Just over half of these BACs were in the break BAC group, comprised of BACs with both ends mapped to the human genome, but to different human chromosomes. This group of BACs is expected to have a high false position rate and, unsurprisingly, the locations of the ends of the whole group of break BACs did not correspond well with inter-chromosomal break points identified during the later construction of the virtual sheep genome (see below). This suggests that most of these are not genuine broken BACs that identify sites of inter-chromosomal rearrangements between the human and sheep genomes. It is quite likely that most of the break BACs in the MegaBAC analysis that are tail-to-tail in the cow and/or dog group resulted from a failure in the coordinate conversion process from the dog and cow genomes to the human genome for at least one end of the BAC. This process was used on the coordinate conversion files generated by the University of California Santa Cruz (UCSC) [28], but we modified the liftOver process to maximize the number of regions that could be lifted over by moving outside of the region of the original match to the cow or dog genome if the original region was not lifted over. Clearly, this process may have also have introduced some incorrect positions in the location of BAC ends that otherwise were unpositionable on the human genome.

Just under half of the tail-to-tail BACs from the cow and/or dog genomes that were not included in the MegaBAC analysis tail-to-tail group were included in the MegaBAC head-to-tail and head-to-tail outsize groups (Table 2). The tail-to-tail BACs from dog and/or cow were enriched in the clustered tail-to-head and tail-to-head outsize groups (Table 2), supporting the proposal that many of these BACs are likely to reflect rearrangements in the human genome relative to the dog, cow, and sheep genomes. In contrast, the head-to-head and head-to-head outsize BACs were under-represented in the clustered groups (Table 2), reinforcing the suggestion that many of the BACs in these groups are not correctly positioned in the MegaBAC analysis.

### Construction of the virtual sheep genome

To construct the virtual sheep genome, the 1,172 cMegaBAC-CGCs need to be reassembled into their predicted locations and orientations on the sheep genome. Of the markers on the public Sheep Map v4.6 (and 11 additional unpublished markers), DNA sequence information is available for only 1,220 different locations, and of these 1,178 could be positioned on the human genome, including a small number in the gaps between cMegaBAC-CGCs and on the Y chromosome. Because relying solely on the markers would not allow all of the cMegaBAC-CGCs to be unambiguously located and fewer still to be oriented, a hierarchical approach was undertaken. Initially, the cMegaBAC-CGCs were linked to each other and their relative orientations determined using tail-to-tail outsize, tail-to-head, tail-to-head outsize and so on BACs with end-sequences located close to the ends of the cMegaBAC-CGCs. Then, the BAC-CGCs built on the cow and dog genomes that linked otherwise unlinked cMegaBAC-CGCs on the human chromosomes were used to reduce further the number of genome segments containing linked cMegaBAC-CGCs (Figure 2). These segments were then anchored to the sheep linkage map using the markers that mapped to the human genome. Forty-six per cent of the cMegaBAC-CGCs, covering 72% of the virtual sheep genome, were positioned using linkage markers and BAC derived linkages to other higher scoring cMegaBAC-CGCs (Table 3).

**Table 3:** Parameters used to classify the accuracy of the position and orientation of the BAC-CGCs on the virtual sheep genome.

Code	Color^a^	Anchored by	Oriented by	% of cMegaBAC-CGCs	% of sequenced genome^a^
1	Dark green	Multiple linkage markers	Multiple linkage markers	15.4%	46.7%
2	Pale green	One linkage marker	Linked directly or indirectly to oriented CGCs by BACs, or part of an oriented block of linked CGCs	5.2%	5.9%
3	Light yellow	Linked directly or indirectly to anchored CGCs by BACs	Linked directly or indirectly to oriented blocks by BACs, or part of an oriented block of linked CGCs	6%	3.2%
4	Dark yellow	Single linkage markers or linked directly or indirectly to anchored CGCs by BACs	Linked directly or indirectly to oriented blocks by BACs, or part of an oriented block of linked CGCs or chicken-mammal or mammalian or cow/human conserved synteny	19.3%	16.4%
5	Orange	Cytogenetic markers or linked directly or indirectly to anchored CGCs by chicken-mammal or mammalian conserved synteny	Chicken-mammal or mammalian or cow/human conserved synteny	29.3%	11.0%
6	Pink	Linked directly or indirectly to anchored CGCs by cow/human conserved synteny or unbroken gene	Chicken-mammal or mammalian or cow/human conserved synteny	13.6%	5.5%
7	Red	Not linked directly or indirectly to an anchored CGC	Orientation uncertain	11.3%	2.7%

^a^based on human Golden path length of 2.84 Gb, excluding Y chromosome. BAC, bacterial artificial chromosome; CGC, comparative genome contig; Gb, gigabases.

A number of regions of conserved synteny between mammals and chickens [22] and within the mammals have been identified [23]. We assumed that within these regions there was a very high probability that the sheep genome would also exhibit conserved synteny with the other mammals. However, these data do not provide orientation or information on BAC-CGC ordering within regions. We used the regions of conserved synteny between cow and humans to order and orient at this level (Table 3). Given the close relationship between sheep and cow, it is likely that there is substantial synteny between the two genomes. During this whole process attention was paid to the genes, and unless there was evidence to the contrary genes were not disrupted during this process. Finally, in the absence of any other information, cMegaBAC-CGCs were positioned using order and orientation on the human genome, minimizing rearrangements between the two genomes. Only 11.3% of the cMegaBAC-CGCs, covering less than 3% of the virtual sheep genome, were in this category (Table 3).

Seven BAC-CGCs constructed on the cow genome and two on the dog genome linked cMegaBAC-CGCs located on different human chromosomes. However, on closer inspection, although the dog inter-chromosomal links appeared to be genuine, five of the seven links predicted from the cow BAC-CGCs appeared to be artefacts caused by the use of an early draft assembly of the cow genome. Thus, the final construction of the sheep chromosomes from the segments of human genome was based predominantly on the order of sheep markers. A more detailed analysis of the predicted junction points between different human chromosomes was undertaken after the construction of the virtual sheep genome. If the junction points were conserved in the cow and/or dog genomes, then the two species specific BAC-CGCs flanking the junction points should be in sequential order. The virtual sheep genome contains 39 junctions between segments located on different human chromosomes, of which only six were supported by the dog BAC-CGCs (including the two links previously identified) and 24 by the cow BAC-CGCs, including the two links previously identified and the six links identified using the dog genome. The five known sheep specific inter-chromosomal junctions relative to cow were also identified. Ten junctions were not supported by the cow BAC-CGC numbering, or were ambiguous, but again this is probably due to the use of an early draft assembly of the cow genome, rather than further sheep specific inter-chromosomal junctions. Alternatively, these junctions might reflect recent rearrangements in the sheep lineage.

**Figure 5:**
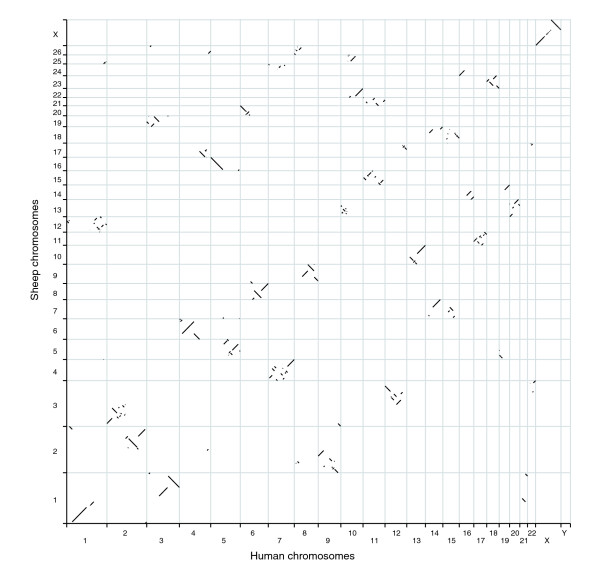
Oxford grid of sheep chromosomes versus human chromosomes. The relationships are plotted at the level of the cMegaBAC-CGCs. BAC, bacterial artificial chromosome; CGC, comparative genome contig.

The full set of relationships between the segments of the sheep and human genomes are shown as an Oxford Grid (Figure 5). Some sheep chromosomes (OAR) such as OAR8 and OARX have a small number of syntenic blocks and are predicted to be very similar in gene order to the equivalent human chromosomes (Table 4). In contrast, other chromosomes such as OAR3 and OAR13 have a large number of syntenic blocks and are predicted to have a large number of rearrangements with respect to their human equivalents (Table 4). The ratio of BAC-CGCs to syntenic blocks is also highly variable, with OARX having a very high ratio reflecting the low BAC coverage (probably because of the high density of repetitive elements and the use of a male animal as the source of DNA for the BAC library) and therefore a large number of BAC-CGCs (Table 4). OAR11 and OAR13 had very low ratios, reflecting substantial reorganization of these segments of the mammalian genome since the last common ancestor of sheep and man. The predicted sizes of the chromosomes were compared with the linkage map and idiogram lengths of the chromosomes (Table 4). Overall, the cytogenetic and sequence lengths were more similar than either was to the linkage map lengths. Removing the unsequenced regions of the human genome from the calculated lengths of the sheep chromosomes (Table 4) had little effect on the fits (data not shown).

**Table 4:** Sheep chromosome details

Chromosome	HSA	CGCs	Synteny blocks^a^	Markers	Mb	Minus gaps (Mb)^b^	Map length (cM)^c^	Cytogenetic Mb^d^
OAR1	1, 2, 3, 21	102	9	119	304.1	276.3	324.4	294
OAR2	1, 2, 4, 8, 9	86	21	102	277.8	259.2	301.3	261
OAR3	2, 9, 12, 22	101	35	106	274.3	251.3	303.2	246
OAR4	7	46	10	41	126.6	126.6	147.8	135
OAR5	1, 5, 19	51	16	39	121.1	117	158.9	126
OAR6	4	37	6	56	123.1	120	155.7	126
OAR7	5, 14, 15	19	9	49	114.8	96.4	148.9	117
OAR8	6	19	3	39	96.8	96.8	128	111
OAR9	6, 8	44	5	42	114.7	111.6	126.9	105
OAR10	13	28	6	27	114.1	95.8	100.2	99
OAR11	17	41	21	37	78.8	78.8	109.6	87
OAR12	1	34	16	31	92.7	82.4	106.4	96
OAR13	10, 20	45	22	32	102.9	98.8	128.3	99
OAR14	16, 19	42	10	39	87.3	78.2	120	84
OAR15	11	48	9	41	85.3	82	123.8	96
OAR16	5	15	2	31	80.6	77.5	84.7	84
OAR17	4, 12, 22	40	11	45	84.7	84.7	130	84
OAR18	14, 15	43	17	36	98.9	80.3	127.7	84
OAR19	3	21	6	28	65.3	65.3	72.1	75
OAR20	6	14	6	41	60.2	58.6	103.3	69
OAR21	11	32	7	19	49.1	49.1	75.5	63
OAR22	10	12	5	18	54.5	54.5	82.9	63
OAR23	18	17	4	40	76.1	74.7	85	78
OAR24	7, 16	26	10	30	70.5	62.4	102.1	63
OAR25	1, 10	26	8	26	54.4	52.3	68.3	63
OAR26	3, 4, 8	19	9	19	55.4	53.9	71.1	57
OARX	X	164	2	40	154.8	152.9	131.3^5^	144

Total		1,172	285	1,173	3,018.9	2837.3	3,657.4	3,000

^a^Number of sets of cMegaBAC-CGCs with consecutive numbers and same orientation as in the human genome. ^b^Predicted lengths of chromosomes after removal of large gaps, >1 Mb, in the human genome sequence. ^c^Map lengths calculated from Sheep Map 4.6 using the sex-averaged lengths, except for the X chromosome where the length of the female map was used. ^d^Chromosome lengths calculated from the sheep ISCNDB (2000) standard cytogenetic maps [47], assuming a genome size, excluding the Y chromosome, of 3 Gb. BAC, bacterial artificial chromosome; CGC, comparative genome contig; Gb, gigabases; HSA, human chromosome; Mb, megabases; OAR, sheep chromosome.

During the construction of the virtual sheep genome, the reordering and reorientation of the cMegaBAC-CGCs changed the group to which 4,266 of the BACs belonged. Of this set of BACs, about 40% were BACs broken in the rearrangement (both ends of the BAC on the same human chromosome, but on two different chromosomes in the virtual sheep genome); of these only about 13% were part of clusters of two or more BACs, In contrast, of the 532 tail-to-tail BACs created by the rearrangements, almost 98% were in clusters of two or more BACs.

### Annotation of the virtual sheep genome

To allow users of the virtual sheep genome browser to be able to assess rapidly the reliability of the location and orientation of the BAC-CGC-defined blocks, they have been color coded from green through to red (Figure 3b). In addition, all of the BAC end mapping coordinates have been transferred to the virtual sheep genome coordinates. To enable users to identify the site and extent of local rearrangements in the human genome relative to the carnivore/ruminant lineage, the coordinates of the dog net tracks form the human genome browser [29] have been converted to the virtual sheep genome coordinates (Figure 3b).

To maximize the utility of the genome to the sheep research community, annotation of features is required. Because it is likely that the general structure of many genes is conserved between sheep and humans [30], the coordinates of the human genes were simply transposed into the coordinates of the virtual sheep genome and an annotation track generated. Other features of the human genome, such as repeat elements, are expected to be very different between the two genomes and therefore were not transferred. A number of features of the BAC end sequences themselves, such as microsatellites, were also transferred to the genome coordinates. The BAC end sequences in the tail-to-tail and tail-to-head BACs (2% false position rate) contain 15,059 predicted microsatellites distributed across the entire sheep genome.

### Identification of candidate BACs to fill the gaps between the cMegaBAC-CGCs

The assembly of a complete sheep BAC tiling path, and later the genome sequence, requires that the gaps between the cMegaBAC-CGCs are filled to generate as small a number of contigs for each chromosome as possible. A number of the gaps are joined by BACS that are head-to-tail, head-to-tail outsize, and so on. We decided to utilize BACs with only one end sequence mapped to the bovine genome to identify BACs that might span the remaining gaps in the assembly. We carried out a much more restrictive BLAST search than in the MegaBAC analysis to generate a set of BACs with only one end mapped to the cow genome (unpaired-in-cow), but in which the confidence of the match was much greater than in the original low stringency BLAST search. The 72,618 BACs with only one BAC end sequence mapping to the cow genome were lifted over to the human genome. The BACs included as tail-to-tail paired end BACs in the MegaBAC analysis were discarded. Of this set of 16,950 BACs, 16,485 mapped to the same position in the human genome from both the unpaired-in-cow and the MegaBAC analyses. The false position rate of 2.7% suggests that the accuracy of the mapping of the rest of the unpaired end BACs may also be very good. The remaining 55,668 BACs were then lifted over to the virtual sheep genome and divided into two groups: those with single hits to the cow genome (high [higher confidence]) and those with multiple hits to the cow genome (low [lower confidence]) with the BLAST parameters used.

These sets of BACs are displayed on the virtual sheep genome browsers with a dotted line of 184 kb (the average length of the BACs in the library) indicating the most probable extent of the BAC in the virtual genome (Figure 4c). An example of a region with a large number of short BAC-CGCs is shown in Figure 4c to illustrate the potential coverage across the gaps in this region. The large number of short BAC-CGCs containing a small number of BACs, few sheep markers, and few predicted human genes are typical of the regions of low confidence in the virtual sheep genome.

### Assessing the accuracy of the predicted locations of the BACs using independent assignments

Unfortunately, only a very small number of BACs from the CHORI-243 library have positions on the virtual sheep genome determined using other methodologies. However, a group of 16 BACs has been mapped to the sheep cystic fibrosis transmembrane conductance regulator gene (CFTR) locus using 'overgo' probes [15] and subsequently sequenced [31]. In our analysis, the positions, order, orientation, and overlaps of the eight tail-to-tail BACs determined from the BAC end sequences were consistent with the positions determined from the more complete sequencing approach. As expected, our analysis did not position any of the other eight sequenced BACs as tail-to-tail BACs anywhere else on the genome. A similar result was obtained with a set of 16 partially sequenced BACs located in the sheep major histocompatibility complex (MHC) region (Groth D, personal communication), of which ten were present in our tail-to-tail set, and three more in our tail-to-head set in equivalent positions. These sets of BACs also contained a number for which we had predicted positions based on the unpaired hits in the cow genome. Of the seven BACs in the CFTR locus, and single BACs in the MHC, agouti signaling protein gene (ASIP) (Norris B, personal communication), and synaptopodin gene (SYNPO) loci, all predictions based solely on the single BAC end sequences were consistent with the positions determined by the more comprehensive sequence analyses. Although only a small sample of BACs, these observations do not contradict the low false position rate of 2.7% observed for the subset of BACs with unpaired BAC-ends in the cow that were included in the tail-to-tail paired BACs in the MegaBAC analysis.

Thirty-six of the BAC end sequences from the library have been positioned on the sheep genome using microsatellites identified in their sequences (Maddox JF, McEwan J, unpublished data). Twenty-eight of the BAC ends are from tail-to-tail BACs in the MegaBAC analysis, five are from BACs with breaks, and three are unpaired. There is only one discrepancy between the predicted and observed positions in the sheep genome for the 36 BAC ends mapped (for the breaks, one end is consistent with the microsatellite mapping), and that BAC end sequence is in a broken BAC, suggesting uncertainty in the mapping to the human genome. Of one BAC at the MHC locus (Groth D, personal communication) and four BACs at the ASIP locus (Norris B, personal communication) that are in the break group, one end of three of the five BACs is in the expected location.

### Comparison of BAC end sequencing and bioinformatics versus fingerprinting and limited BAC end sequencing

The cow BAC fingerprinting project undertaken by the International Bovine BAC Mapping Consortium [2] grouped 257,914 cow BACs into 655 BAC contigs (1 May 2006 release [32]) and a much larger number of singletons.

**Figure 6:**
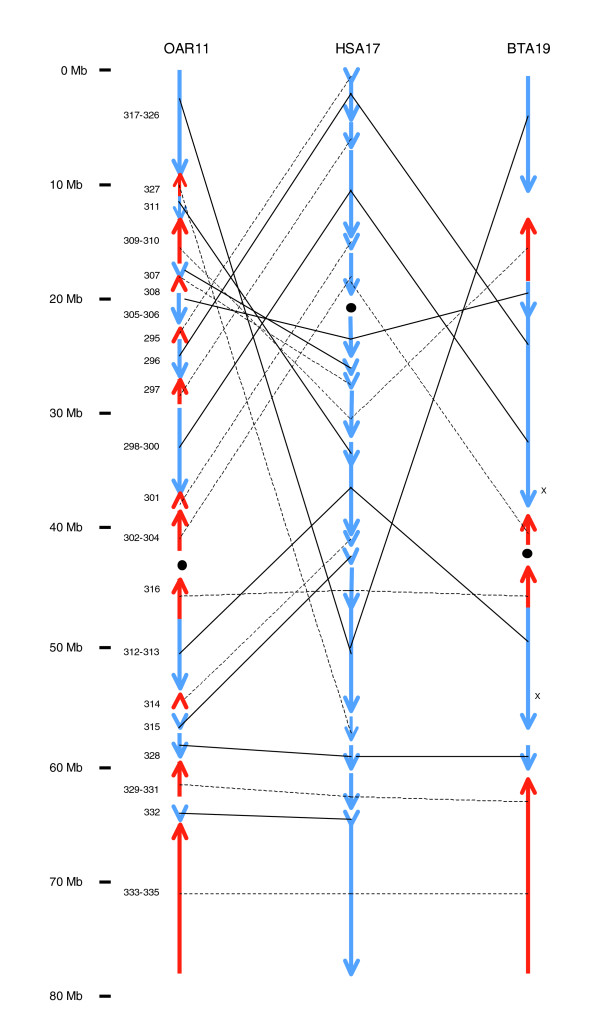
Relationship of chromosomes OAR11 and BTA19 to HSA17. The segments of HSA17 corresponding to groups of BAC-CGCs predicted to be syntenic are shown by blue vertical lines with an arrowhead at one end, in the order in which they occur in the human genome. The arrowheads in the map of OAR11 show the order and the orientation of the syntenic segments in the sheep genome with respect to the human genome: blue, same orientation; and red, reversed orientation (with Mb scale on the left). The solid lines link syntenic segments with the same orientation in the sheep and human genomes and the dotted lines link syntenic segments inverted in the sheep genome relative to the human genome. The larger black dot indicates the location of the human centromere and the predicted location in the other genomes. The numbers to the left of the syntenic segments indicate the IDs of the cMegaBAC-CGCs built on the human genome. On the far right is shown an equivalent representation of BTA19 built using BAC fingerprinting and limited BAC end sequencing data, adapted from Everts-van der Wind and coworkers [11]. Two small inversions in the cow assembly identified from the BAC end sequencing are indicated by 'x'. BAC, bacterial artificial chromosome; BTA, cow chromosome; CGC, comparative genome contig; HSA, human chromosome; Mb, megabase; OAR, sheep chromosome.

The BAC fingerprinting and a portion of the cow BAC end sequencing data have been combined for one cow chromosome (BTA), namely BTA19 (Figure 6), which is the ortholog of HSA17 [33] and OAR11. Twenty-two BAC contigs were anchored to BTA19, covering about 60% of the chromosome [11]. For the equivalent sheep chromosome (OAR11) the initial 41 cMegaBAC-CGCs covered 88% of the chromosome. Using additional linkage information contained in the BAC end sequence mapping data, these were reduced to eight linked groups of two or more cMegaBAC-CGCs (77.5% of the total chromosome) and 13 unlinked cMegaBAC-CGCs (10.5% of the total chromosome). Thus, for these equivalent chromosomes, our approach has covered more of the chromosome in a similar number of blocks to the combination of fingerprinting and limited end sequencing. Comparison of chromosome maps reveals a very high level of congruency in the location of breakpoints and rearrangements between OAR11 and HSA17 and between BTA19 and HSA17 (Figure 6).

## Discussion

Clearly, for the purposes of constructing a set of BAC-CGCs with maximum coverage of the sheep genome, we needed to maximize the number of aligned BAC clones with paired ends. Our approach, combining hits from several species onto the framework of one genome, has achieved a very high hit rate of sheep BACs mapped to the human genome, generating linked and single sheep cMegaBAC-CGCs. The two sets of the tail-to-tail BACs and unpaired-in-cow BACs include predicted positions for 140,292 of the 192,998 BACs (72.7%), with a low rate of false positions. By comparison with our results, a published analysis using a set of cow BAC end sequences against the human genome with much more conservative mapping parameters contained only about 4% of paired end BACs aligned with the human genome [34]. A more recent analysis with a small set of horse BAC end sequences against the human genome included about 17.5% of the BAC end sequences in paired end BACs aligned with the human genome [35].

The higher success rate in our study is most likely due to a combination of the length and quality of the paired end reads, the high proportion of clones with paired end reads, optimized alignment parameters, enhancements to the reference human genome assembly, the strategy of combining data from multiple genomes, the use of the UCSC genome coordinate conversion files, and the development of the 'pseudo-liftover' strategy to maximize the coordinate conversion process. As a consequence, more than 70% of the virtual sheep genome length is contained within cMegaBAC-CGCs that have been positioned directly or indirectly using the mapped sheep markers. The utilization of several sets of vertebrate synteny data further increased the proportion of BAC-CGCs positioned on the virtual sheep genome, albeit at a lower level of confidence. Clearly, there are still a significant number of genes located in the less certain cMegaBAC-CGCs, although many of these appear to contain gene sparse regions, and in the gaps between cMegaBAC-CGCs that are not adjacent in the human genome, where arbitrary junctions have been made. Users of the resource must be cautious interpreting the locations of genes in these regions. Our strategy also enabled us to maximize the use of the cow genome sequence, although it was only available as an early draft assembly at the time when this analysis was undertaken.

We have taken a pragmatic approach to the generation of the first version of the virtual sheep genome, aiming to use as much relevant data as possible to generate as accurate a map as feasible, but also with maximum coverage of the genome. The virtual sheep genome covers almost all regions of the sheep genome othologous to the human genome. By identifying and classifying the regions of uncertainty and providing access to the various types of mapping data, interested users can make their own decisions about the reliance that they wish to place on the information. In doing this we have taken the risk of 'humanizing' the sheep genome, because we believe that users of the genome prefer to sacrifice some accuracy for more comprehensive coverage. Clearly, as more genomes are released, the accuracy of the map will be increased significantly. A number of sheep radiation-hybrid (RH) panels have been constructed [36], and we plan to anchor representative BACs from each of the cMegaBAC-CGCs to the sheep genome by RH mapping in order to validate the order and orientation of the cMegaBAC-CGCs in the virtual sheep genome, in particular the accuracy of the additional methods of assigning cMegaBAC-CGCs using conserved vertebrate and mammalian synteny.

The resolution of the map varies, and in some positions using the information provided on the virtual sheep genome browser allows sites of rearrangement (with respect to the human genome) to be localized to within very small segments of the genome. However, because genome rearrangements occur at all resolutions, and it has been reported that there are more inversions and rearrangements of less than 1,000 bases to more than 1,000 bases between the human and mouse genomes [24], this is a fairly low resolution map of the sheep genome. However, using the definitions of Pevzner and Tesler [25], we certainly identify many micro-rearrangements (those less than 1 megabase in length). For the utilization of SNP based whole genome scans and gene 'discovery', accuracy at the 1,000 base level is unlikely to be required because many, if not most, of these will not affect the positioning of genes.

Across the virtual sheep genome, excluding the sex chromosomes, there are 285 sheep/human syntenic blocks. This is rather larger than the figure of 159 blocks reported between the human and dog genomes (a comparable pair of species) based on 1.5× genome survey sequencing of the dog genome [8]. However, it is in the range of the 275 dog/human segments that are greater than 100 kb, and the 348 human/dog segments that are greater than 100 kb calculated from the UCSC genome sequence alignment nets [37]. Equivalent figures for segments greater than 300 kb are 221 and 231, respectively [37]. Because the number of blocks identified is dependant on the way in which they are calculated [17], we also calculated the number of dog/human syntenic blocks based on the sheep BAC mapping. The number of 259 blocks (excluding the sex chromosomes) indicates that the resolution of the virtual sheep genome is probably between 100 and 300 kb, and suggests that we have not significantly over-clustered or under-clustered BACs into cMegaBAC-CGCs in the construction of the virtual sheep genome. However, a small number of significant discrepancies between the position of markers on the sheep map and their predicted location on the virtual sheep genome were identified. Although most of these appeared to be due to problems with positioning the sheep markers on the bovine genome, or in the liftover from the bovine to the human genome, a small number of these markers appeared to identify a very low level of over-clustering of the BACs. Seven possible such over-clustering events, in which three or more BACs that are not in the tail-to-tail group also support the sheep map marker positions, have been identified. A selection of BAC end sequences from both sections of the seven cMegaBAC-CGCs will be positioned on the sheep map to confirm, or otherwise, the apparent over-clustering of BACs.

While this work was underway, the construction of a similar sized sheep BAC library was described [38]. It is possible that this may complement the CHORI-243 library. However, the locations of the gaps in the assembly suggest that the size of the library *per se *was not a limiting factor in the construction of the virtual genome. The alignment of many of the gaps between cMegaBAC-CGCs with the gaps between the regions of chicken-mammal conserved synteny (Figure 3) and regions of rearrangements between the dog and human genomes suggests that the purely bioinformatics approach may be limited by the presence of regions with high levels of rearrangements in one or more of the genomes. These regions would not pose such problems for a targeted BAC fingerprinting project aimed at closing the remaining gaps in the assembly. Here, the problem is to identify efficiently limited sets of BACs to fingerprint. Our analysis of the false position rates shows that using the tail-to-head and unpaired-in-cow groups of BACs as a source of BACs in the gaps between cMegaBAC-CGCs is likely to generate sets with an acceptably low number of incorrectly positioned BACs. If more BACs are required, then clustered BACs in the tail-to-tail and tail-to-head outsize groups, and then the head-to-head BACs, should be used. Finally, the break BAC group, with a 65% false position rate based on a limited set of ten BACs, should be used.

## Conclusion

The BAC-end sequences themselves provide a source of sequences for the identification of sheep SNPs, and a re-sequencing project is underway as part of the development of a SNP chip for genotyping sheep. In addition, a number of the new microsatellites has already been validated (Maddox JF, McEwan J, unpublished data). The virtual sheep genome will play a major role in the identification of appropriate markers and in the interpretation of the results of whole genome scans. Furthermore, the analysis allows the selection of BACs for a minimal tiling path across the majority of the sheep genome, provides a strategy to close the gaps in the tiling path, and for almost all sheep genes predicts BAC(s) that are likely to contain the gene. Thus, the virtual sheep genome is an essential tool for sheep researchers undertaking genomic and genetic experiments in sheep.

Our comparative genomics approach can generate BAC contigs comparable to using BAC fingerprinting alone. In addition, unlike BAC fingerprinting, the method also provides a prediction of the gene content of more than half of the BACs in the library and almost all genes in the genome, as well as a prediction of gene order across the genome, providing an alternative to oligonucleotide based hybridization methods [14,15]. With the high level of assignment of BACs, this approach is also competitive with pooled genome indexing and even ST-pooled genome indexing strategies [13].

The approach can be applied to any genome and requires a set of paired end sequence reads with high genome coverage (in this example, a BAC library). It also requires a well assembled reference genome sequence from a related species (in this example, the human genome). The less related the reference species, the larger the number of BAC-CGCs will be, and the greater the number of markers required to order and orient the segments. Including the sequences of additional genomes will increase the number of BACs positioned on the reference genome and reduce the number of BAC-CGCs. These genome sequences can be drafts, but to make full use of the strategy they should be assembled into sequence contigs and scaffolds (cow in this example), even if they are not ordered and oriented, let alone assigned to chromosomes.

The inclusiveness of the final virtual genome will depend on a number of factors, including the marker density of the species map and the extent to which reliance is placed on other sources of information, for example conserved order of segments on the scaffolding genomes and the regions of conserved synteny across the mammals. However, given our experience with the sheep genome, for mammals with a large BAC library and around 1,000 markers, a substantial proportion of the genome - and a greater proportion of the protein coding genes - will be contained in anchored and oriented BAC-CGCs.

## Materials and methods

### Construction and characterisation of the sheep BAC library

The preparation of the CHORI-243 sheep BAC library followed the cloning approach as previously described [39] from the blood of a Texel breed ram (animal number 200118011, MARC population). DNA was isolated from white blood cells by embedding the cells in agarose. Agarose-embedded DNA was partially digested with a combination of *EcoR*I restriction enzyme and *EcoR*I methylase, and the fragments were size fractionated by pulsed field gel electrophoresis into five fractions in the range 150 to 250 kb. DNA fragments in the overlapping ranges 180 to 220 kb and 200 to 250 kb were cloned into the pTARBAC2.1 vector between the *EcoR*I sites. The ligation products were then transformed into DH10B (T1 resistant) electro-competent cells (Invitrogen, Carlsbad, CA, USA). The library was arrayed into 384-well microtiter dishes and subdivided into two segments; segment 1 included plates 1 to 240, and segment 2 included plates 241 to 528. It was also gridded onto eleven 22 × 22 cm nylon high-density filters for screening by probe hybridization. Each hybridization membrane represents more than 18,000 distinct sheep BAC clones, stamped in duplicate. Data on the CHORI-243 clone average insert size was determined by pulsed field gel electrophoresis from 528 clones. While analyzing clones using pulse field gel electrophoresis to determine the average insert size, a small number of noninsert clones (2.1%; clones containing a small deleted vector fragment consistent with sucrose resistance) were recorded (Figure 1).

### BAC end sequencing and analysis

Transformed bacteria were grown with rotation (510 rpm) for 20 hours at 37°C in 384-deep-well plates containing 200 μl medium per well. Cells were pelleted, and DNA purified by alkali lysis and precipitation. Routinely, five random samples from each plate of templates were examined by agarose gel electrophoresis for appropriate DNA content before DNA sequencing. Sequencing reactions were conducted in 384-well plates using T7 or SP6 primers, and Big Dye Terminator Sequencing Mix (Applied Biosystems, Foster City, CA, USA). Thermal cycling at 96°C (10 s), 54°C (5 s), and 60°C (4 min) was conducted for 120 cycles. Following isopropanol precipitation, the DNA was analyzed using ABI 3730 Sequencers. For successful reads (>50 bases after trimming), complete sequence traces were deposited in the National Center for Biotechnology Information (NCBI) Trace Archive with the following accession numbers: ti:467413973-ti:467420806, ti:901356365-ti:901363259, ti:918696780-ti: 918816778, ti:918828258-ti:918908255, ti:918913846-ti:919053432, ti:953094427-ti:953097258, ti:958315633-ti:958335631, and ti:963889872-ti:963890220. Reads edited to remove vector sequence and low-quality data were deposited in GenBank with the following accession numbers: CL632218-CL639051, CZ920079-CZ926973, and DU169919-DU532729.

The full set of sheep BAC end sequences were analysed for the occurrence of microsatellites using Sputnik [40] (-A -s 16 [minimum score]) and Tandyman [41] (-l 2 [repeat size lower limit] -u 10 [repeat size upper limit] -m 10 [minimum units in repeat]).

### Mapping BAC end sequences to cow, dog, and human genome assemblies and assembly of sheep BAC-CGCs

The full set of sheep BAC end sequences was aligned to the lower case masked versions of the human genome (build hg17) and the dog genome (build canFam2), using BLASTn with the following parameters: -W 7 -r 17 -q -21 -G 29 -E 22 -X 240 -e 1 -f 280 -F m -U T and -z 3076781887(human) and -z 2531657226 (dog). The BAC end sequences were aligned to the lower case masked versions of the cow genome (build Btau2.0), using MegaBLASTn with the following parameters: -U T -D 2 -H 1 -W 16 -e 0.01 -p 60 -F m. The human, dog, and cow genome sequences were obtained from UCSC Genome Bioinformatics site [29,42]. The searches were conducted on the CSIRO Bioinformatics Facility Beowulf cluster, comprised of 66 Dell Blade 1655MC dual PIII processor machines. No cut-offs were applied to the BLAST output except that for each sheep BAC end sequence only the best hit from each of the genomes was used for the next steps. The locations of all sheep BACs with the BAC ends mapping in a tail-to-tail configuration (the two ends in opposing orientations with 3' ends internal) and between 10 kb and 500 kb apart on the genome were retained. BAC-CGCs were constructed for each genome scaffold using Perl scripts to process the data. Starting from the beginning of each chromosome for each of the three species, the first sheep BAC that overlapped with a second sheep BAC was identified, and the BAC-CGC was extended until no further overlapping sheep BACs were identified. This process was repeated along the chromosome until the last sheep BAC located on the chromosome was reached.

To generate the unpaired-in-cow group of BAC ends the full set of sheep BAC end sequences were aligned to the lower case masked cow genome using MegaBLASTn with the following parameters: -U T -D 2 -W 32 -p 80 -e 1e-8 -F m.

### Coordinate conversion and construction of MegaBAC-CGCs

The coordinates from the mapping of the sheep BAC ends to the dog and cow genomes were converted to the framework of the human genome using the liftOver utility [28] and the canFam2 to hg17 and Btau2.0 to hg17 chain files, also downloaded from UCSC genome bioinformatics site [42]. If the initial liftOver was not successful, then regions of 100 bases either side of the BAC end sequence were taken and positioned using liftOver (pseudo-liftOver). If this was again unsuccessful then the process was repeated in steps of 100 bases until a successful liftOver was achieved, or a distance of 10 kb was reached. Positions of the members of the various groups of BACs were determined using a series of Perl scripts. With the exception of the unpaired-in-cow group, any single BAC could only be a member of one group of BACs. BACs were assigned to groups in the MegaBAC analysis in the following priority order: tail-to-tail, tail-to-head, tail-to-head outsize (<10 kb apart or >500 kb apart), tail-to-tail outsize, head-to-head, head-to-head outsize, break, and unpaired. BACs in the tail-to-tail group were excluded from the unpaired-in-cow group (see above). BACs with only one ended sequenced were designated as singletons and could only be in the unpaired-in-cow, unpaired, or unmapped groups. In cases of conflicting mapping to the human genome from the different sources and within the same BAC group, positions determined by mapping via the cow genome had priority over the mapping via the dog genome and both had priority over the direct mapping to the human genome.

To estimate the false position rate the assignment of the BAC end sequences to the BACs was randomized and the mapping data for the human genome were re-run through the BAC pair mapping Perl scripts. The randomization of the BAC ends to the BACs was repeated ten times, and the number of tail-to-tail paired BAC-ends between 10 kb and 500 kb apart was counted for each randomization and averaged. To calculate the rates for the other groups of BACs the calculated values were halved to allow for the approximately 50% of BACs in the real data that were in tail-to-tail paired end BACs and therefore unable to contribute to any other group of BACs.

The MegaBAC-CGCs were constructed on the human genome using the full set of MegaBAC analysis BACs, as described above for the BAC-CGCs built on the cow, dog, and human genomes. Average BAC coverage and average BAC length where calculated along each chromosome at 50 kb intervals.

### Construction of sheep via dog and sheep via cow BAC-CGCs and consolidated MegaBAC-CGCs on the human genome

For each BAC-CGC built on the dog or cow genome, the outermost BACs that had also been included within one or two or more adjacent MegaBAC-CGCs built on the human genome were identified and a new BAC-CGC block drawn between the outermost BAC end sequences. These are the sheep-via-dog BAC-CGC and sheep-via-cow BAC-CGC tracks on the human genome browser [43]. In the cases in which a single BAC-CGC built on the dog or cow genomes contained BACs from two or more unadjacent MegaBAC-CGCs built on the human genome, the BAC-CGCs were split using the innermost BACs flanking the location of the break of synteny. These are the sheep-via-dog, and sheep-via-cow, BAC-CGC breaks tracks on the human genome browser.

Adjacent MegaBAC-CGCs that were linked by sheep-via-dog and/or sheep-via-cow BAC-CGCs were consolidated into a single so-called cMegaBAC-CGC. To generate the dog-versus-human net tracks, the hg17.netCanFam2 table was downloaded from the UCSC Genome Bioinformatics website using the table browser [42] and the data were extracted and converted to GFF format for display on the human genome browser.

### Mapping of sheep markers to the human genome

Sheep markers on the linkage and cytogenetic maps were positioned on the human genome using a number of different approaches. Initially, lower case masked sequences for all markers (for which ovine sequence data were available) were aligned with the lower case masked cow genome Btau2.0 using BLASTn (-e 1e-5 -U T and the rest default parameters) and the top high scoring pair for the top hit retained. Sequences not aligned using this approach were then aligned unmasked to the cow genome using BLASTn (-e 1e-5 and the rest default parameters), increasing the number of hits by just under 10%. The cow genome coordinates were then converted to human genome coordinates using the LiftOver program [28] and the Btau2.0 to hg17 chain file as described above. Where conversion of the match coordinates was not successful, the pseudo-liftOver strategy described above was used.

### Construction of dog/human synteny blocks

The dog/human synteny blocks were constructed by scanning the sheep-via-dog BAC-CGCs positioned onto the human genome coordinates for sequentially numbered blocks with the same orientation. Single missing BAC-CGCs were allowed, but two or more missing blocks in a row triggered the end of a region of conserved synteny exclusive of the missing blocks.

### Chicken-mammal, mammalian, and cow-human conserved synteny

The regions of chicken-mammal conserved synteny were taken from the report by Bourque and coworkers [22]. The regions of mammalian conserved synteny and cow-human conserved synteny were taken from the report by Murphy and colleagues [23]. The locations were converted from hg15 to hg17 coordinates using the liftOver utility (see above) on the hg15-to-hg16 and hg16-to-hg17 chain files. For successful conversion of the coordinates of such large regions over two versions of the human genome assembly, the two ends of each block (1 kb in size) were converted separately. Only two blocks failed to map both ends using this technique; these two blocks were subsequently mapped to hg17 using BLAT.

### Construction and annotation of the sheep virtual genome

To generate the virtual sheep genome the mid-point between each pair of cMegaBAC-CGCs was identified. If the mid-point was located in a gene (NCBI human RefSeq mRNAs were used to define the extent of a gene), then the position closest to the mid-point and not in a gene was identified. The flanking BAC-CGCs were then extended to this point or, in the case of the first and last BAC-CGCs on a human chromosome, to the start or end coordinate of the chromosome. Thus, all nucleotides in the human genome sequence were included in a block and therefore the virtual sheep genome is exactly the same length as the human genome. A liftOver chain file was constructed for each sheep chromosome that mapped the human hg17 coordinates of the start and end positions of each extended BAC-CGC to the virtual sheep genome coordinates. Using the liftOver utility and the virtual sheep genome chain file, the BAC end and BAC-CGC mapping coordinates, and any features mapped to the human genome, were converted to the virtual sheep genome coordinates.

### Access to data

The mapping of the sheep BACs to the human, dog and cow genomes, the BAC-CGCs, and other datasets generated during the course of the analysis and the virtual sheep genome are available from the livestockgenomics website [44]. All of the data are displayed via interactive genome browsers built using Gbrowse [45].
